# Comparison of Prognosis and Metachronous Gastric Tumor Rates After Endoscopic Submucosal Dissection Between Gastric Neoplasm of Fundic Gland Type Neoplasms and Conventional Gastric Adenocarcinoma

**DOI:** 10.7759/cureus.58467

**Published:** 2024-04-17

**Authors:** Junnosuke Hayasaka, Shu Hoteya, Yugo Suzuki, Yorinari Ochiai, Yutaka Mitsunaga, Hiroyuki Odagiri, Akira Masui, Daisuke Kikuchi, Yutaka Takazawa

**Affiliations:** 1 Gastroenterology, Toranomon Hospital, Tokyo, JPN; 2 Pathology, Toranomon Hospital, Tokyo, JPN

**Keywords:** metachronous tumor, prognosis, oxyntic gland adenoma, gafg, fundic gland type

## Abstract

Introduction: Gastric neoplasm of the fundic gland type (GNFG) is a tumor with a good prognosis. However, since it has not been compared with conventional gastric adenocarcinoma (CGA), it is unknown whether it has a good prognosis or requires surveillance after treatment. The purpose of this study was to determine the prognosis and metachronous gastric tumor rates compared with those of CGA.

Methods: We conducted a single-center, retrospective, matched-cohort study using our database from January 2010 to December 2021. We extracted GNFG data from the endoscopic submucosal dissection (ESD) database and matched patients with conventional early gastric cancer as controls in a 1:4 ratio by age and sex. GNFG and CGA were compared for the overall survival (OS), disease-specific survival, progression-free survival, and metachronous gastric tumor rates.

Results: Overall, 43 lesions were GNFG and 164 CGAs were matched. There were three deaths in the GNFG group and 11 deaths in the CGA group. There was no significant difference in the OS between the two groups (P=0.81). The five-year OS rates for the GNFG and CGA groups were 90.9% and 92.9%, respectively. No disease-specific deaths or recurrences were observed in either group. There was no significant difference in the cumulative metachronous gastric tumor rate between the two groups (P=0.17). The cumulative five-year metachronous gastric tumor rates for the GNFG and CGA groups were 6.6% and 2.5%, respectively.

Conclusions: The prognosis for GNFG is good, however, not better than that for CGA. The metachronous gastric tumor rate after ESD in GNFG was not lower than that in CGA. Therefore, after ESD, GNFG may need to be managed in the same way as CGA.

## Introduction

Gastric adenocarcinoma of the fundic gland type (GAFG) was first reported in 2010 as a new type of gastric cancer [1], and many reports on GAFG have since been published [2-8]. GAFG resembles a fundic gland with mild nuclear atypia [1]. GAFG is characterized by a high rate of submucosal (SM) invasion even when the tumor is small [1,4]. GAFG is a gastric adenocarcinoma that is not associated with *Helicobacter pylori* (HP) infection [1,4]. In the 2019 World Health Organization (WHO) classification, GAFG was introduced as a new gastric adenocarcinoma [9]. GAFG is considered to have low malignancy and a good prognosis because of its low nuclear atypia, low desmoplastic reaction even when SM invasion is present, and almost no lymphovascular invasion or p53 protein overexpression [1,5,8]. Therefore, some reports refer to GAFG as oxyntic gland adenoma (OGA) or polyp [2]. OGA was also introduced into the 2019 WHO classification [9]. In Japan, GAFG was considered as a gastric carcinoma with a special histological subtype in 2017 [10]. However, Ushiku et al. [6] reported in 2020 that a GAFG that remains intramucosal should be considered an OGA, and whether GAFG is a carcinoma is controversial. Therefore, GAFG and OGA were treated as gastric neoplasms of the fundic gland type (GNFG) in this study.

Lymphovascular invasion and advanced cancer have been reported in a few cases of GNFG [7,11,12]. Moreover, there are no reports comparing the prognosis of GNFG with that of conventional gastric cancer. Therefore, it is unclear whether GNFG is truly a low-malignancy tumor with a good prognosis. Furthermore, it is unknown whether surveillance is necessary after endoscopic treatment since the metachronous gastric tumor rate is also unknown.

Therefore, we compared the prognosis and metachronous gastric tumor rate of GNFG with those of conventional gastric adenocarcinoma (CGA) to contribute to future treatment strategies and surveillance of GNFG.

## Materials and methods

We conducted a retrospective matched-cohort study using data from our gastric endoscopic submucosal dissection (ESD) database from January 2010 to December 2021. The database contains 2634 consecutive lesions who underwent ESD for early gastric carcinoma at our hospital. We extracted GNFG data from the database and matched patients with early gastric cancer as controls in a 1:4 ratio by age and sex. If more than five lesions matched, we selected the four lesions closest to the date of ESD for GNFG. If the four cases did not match, one to three lesions were matched. GNFG was defined as a gastric neoplasm resembling fundic-gland cells. CGA was defined as tubular, papillary, poorly cohesive, mucinous, and mixed adenocarcinoma according to the Japanese classification of gastric carcinoma [10]. All patients provided written informed consent for the ESD. This study was approved by the Toranomon Hospital Ethics Committee (approval number: 2382).

The primary endpoint was overall survival (OS). The secondary endpoints were disease-specific survival (DSS), progression-free survival (PFS), and metachronous gastric tumors. Metachronous gastric tumors were defined as gastric lesions discovered one-year post-ESD. Recurrence included local recurrence, lymph node metastasis, and distant metastasis as confirmed by imaging or histology. The following parameters were evaluated: age, sex, body mass index (BMI), performance status (PS) [13], Charlson comorbidity index (CCI) [14], HP status, endoscopic findings, pathological findings, endoscopic curability, additional surgery, and follow-up period. Pathological findings were based on the Japanese Gastric Cancer Association's Cancer Treatment Regulations [10]. Endoscopic curability was defined according to the guidelines for ESD and endoscopic mucosal resection (EMR) for early gastric cancer as follows [15]. Endoscopic curability (eCura) corresponds to curative resection (eCura A), extended curative resection (eCura B), or noncurative resection (eCura C-1/C-2), respectively. eCura A was defined as a lesion resected en bloc and was (1) predominantly differentiated, pT1a (intramucosal cancer), with no ulcerative findings; (2) tumor size <3 cm, predominantly differentiated, pT1a, with ulcerative findings; or (3) tumor size <2 cm, predominantly undifferentiated, pT1a, no lymphovascular invasion, and negative surgical margins. eCura B was defined as a lesion resected en bloc with a tumor size <3 cm, predominantly differentiated type, pT1b (submucosally invasive cancer and cancer invasion <500 μm from the muscularis mucosae), no lymphovascular invasion, and negative surgical margins. eCura C-1 was defined as a lesion in which the only noncurative factor after ESD was piecemeal resection or a positive horizontal margin. However, eCura C-2 was defined as a lesion for which the eCura A/B/C-1 criteria were not met. In eCura C-2, additional surgery is recommended in principle, but not always, depending on the patient's general condition and wishes. Surveillance after ESD was performed according to the gastric cancer ESD/EMR guidelines at that time [15,16]. Surveillance was recommended every 6-12 months for eCura A and B. Biannual surveillance was recommended for cCura C. The follow-up period was from the date of treatment to the date of death or last visit. The endoscopic follow-up period was defined as the date of treatment to the date of the last endoscopic observation.

HP status

HP-currently infected was defined as a positive urea breath test or stool antigen test. HP-eradicated was defined as a negative stool antigen test (Meridian Inc., Cincinnati, United States) or a 13C-urea breath test (Otsuka Pharmaceutical Co., Ltd., Tokushima, Japan) with a clear history of eradication therapy. HP-spontaneously eradicated was defined as a negative stool antigen test or 13C-urea breath test without a clear history of eradication therapy. HP-uninfected was defined as no history of eradication therapy, no endoscopic atrophy, a regular arrangement of collecting venules in the lesser curvature of the gastric angle, and a negative serum HP-antibody test (<3 U/mL (E-plate test; Eiken Chemical Co., Ltd., Tokyo, Japan) or <10 U/mL (*H. pylori*-latex; Denka Seiken Co., Ltd., Tokyo, Japan)).

ESD procedure

For our standard ESD procedure, we used a dual knife through a 2-channel endoscope (2TQ260M; Olympus Corporation, Tokyo, Japan) equipped with an electrosurgical generator (ICC 200 or VIO 300D; Erbe, Tübingen, Germany). We marked the area at least 5 mm from the lesion. After the submucosal injection of a glycerol solution (10% glycerol and 5% fructose; Chugai Pharmaceutical, Tokyo, Japan) containing indigo carmine, a mucosal incision was made, followed by mucosal dissection. After specimen retrieval, the visible vessels on the ESD ulcer were prophylactically coagulated using hemostatic forceps.

Statistical analyses

Continuous variables are expressed as medians and interquartile ranges and were compared using the Wilcoxon rank-sum test. Categorical variables were compared using the chi-square test or Fisher's exact test. OS, cancer-specific survival, PFS, and the incidence of metachronous gastric carcinoma were evaluated using Kaplan-Meier curves and compared using the log-rank test.

Statistical significance was set at P<0.05. All analyses were performed using the R software version 4.2.3 (The R Foundation for Statistical Computing, Vienna, Austria).

## Results

**Figure 1:**
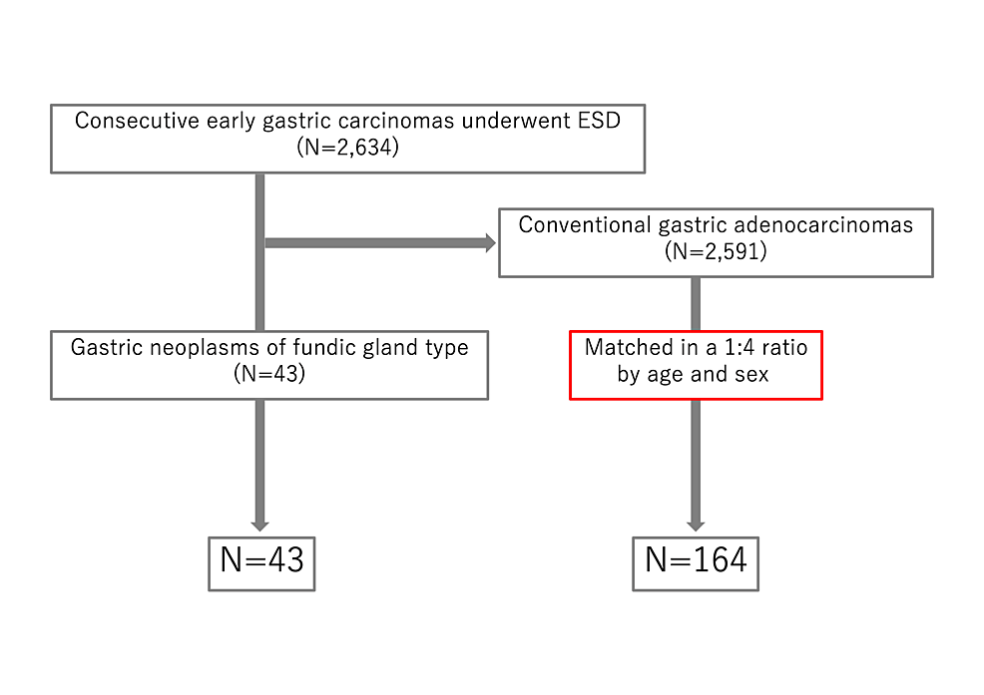
Flow chart of this study

A flowchart of this study is shown in Figure 1. There were 2,634 lesions of early gastric cancer lesions that underwent ESD during the study period. Of these, 43 lesions were GNFG, and 2,591 lesions were CGA. Additionally, 164 CGAs were matched. Background, lesion characteristics, and outcomes are shown in Table 1. There were no significant differences in BMI, PS, or CCI between the two groups. GNFG group had more HP uninfected and less atrophic gastritis. The GNFG group was significantly more common in the upper third of the stomach and had a significantly more elevated type (P<0.001, P<0.001). In the GNFG group, the histological type was a differentiated type in all cases, significantly more than in the CGA group (P=0.026). Tumor size in the GNFG group was significantly smaller (P<0.001). Although SM invasion was significantly more common in the GNFG group than in the CGA group (37.2% vs. 12.2%, P<0.001), SM deep invasion was not significantly different between the two groups (2.3% vs. 3.0%, P=1.0). Ulceration findings were significantly less common in the GNFG group (P=0.033). Lymphatic and venous invasions were not observed in the GNFG group, but the difference was not significant (P=0.35 and P=0.58, respectively). There was significantly lower eCura C-2 in the GNFG group (P=0.019), and fewer lesions were indicated for additional surgery. Of the cases with eCura C-2, one case in the GNFG group and 13 cases in the CGA group underwent additional surgery. There were no residual tumors or lymph node metastases in the surgical specimens from either group.

**Table 1:** Clinical characteristics and outcomes of GNFG and CGA Values are presented as n (%) and median (interquartile range). Continuous variables were compared using the Wilcoxon rank-sum test. For categorical variables, a chi-square test was performed for the legend* and a Fisher's exact test for the others. Statistical significance was set at P<0.05. SM is classified into SM1 and SM2; SM1 is defined as cancer invasion <500 μm from the muscularis mucosae and SM2 is defined as invasion ≥500 μm. CGA: Conventional gastric adenocarcinoma; GNFG: Gastric neoplasm of fundic gland type; IQR: Interquartile range; M: Confined to mucosa; SM: Confined to submucosa

Variables	GNFG group N=43	CGA group N=164	P-value
Age (years)	67.0 (57.5, 76.5)	68.0 (60.0, 77.0)	0.66
Sex (Male)	30 (69.8)	115 (70.1)	1.00*
Body mass index	22.7 (20.7, 24.3)	23.3 (21.0, 25.6)	0.27
Performance status ≥ 2	1 (2.3)	0 (0.0)	0.21
Charlson comorbidity index ≥ 2	9 (20.9)	47 (28.7)	0.41*
*Helicobacter pylori* status			<0.001
Currently infected	1 (2.3)	36 (22.0)	
Eradicated	18 (69.2)	89 (54.3)	
Spontaneously eradicated	8 (18.6)	28 (17.1)	
Uninfected	16 (34.9)	5 (4.8)	
Uncertain	0 (0.0)	6 (3.7)	
Atrophic gastritis	24 (55.8)	158 (96.3)	<0.001*
Open-type	16 (37.2)	125 (76.2)	<0.001*
Location			<0.001
Upper third of the stomach	34 (79.1)	30 (18.3)	
Middle third of the stomach	6 (14.0)	38 (23.2)	
Lower third of the stomach	3 (7.0)	96 (58.5)	
Macroscopic type			<0.001
Elevated	28 (65.1)	39 (23.8)	
Flat	9 (20.9)	11 (6.7)	
Depressed	4 (9.3)	101 (61.6)	
Mixed	2 (4.7)	13 (7.9)	
Histologic type			0.026
Differentiated	43 (100.0)	147 (89.6)	
Undifferentiated	0 (0.0)	17 (10.4)	
Tumor size (mm)	6.0 (4.5, 9.5)	15.0 (9.0, 20.3)	<0.001
Tumor depth			<0.001*
M	27 (62.8)	144 (87.8)	
SM	16 (37.2)	16 (12.2)	
Tumor depth SM2	1 (2.3)	5 (3.0)	1.00
Ulceration findings	1 (2.3)	24 (14.6)	0.033
Lymphatic invasion	0 (0.0)	7 (4.3)	0.35
Venous invasion	0 (0.0)	4 (2.4)	0.58
Horizontal margin	0 (0.0)	1 (0.6)	1.00
Vertical margin	0 (0.0)	5 (3.0)	0.59
Endoscopic curability			<0.001
A	27 (62.8)	137 (83.5)	
B	15 (34.9)	1 (0.6)	
C-1	0 (0.0)	1 (0.6)	
C-2	1 (2.3)	25 (15.2)	
Endoscopic curability C-2	1 (2.3)	25 (15.2)	0.019
Additional surgery	1 (2.3)	13 (7.9)	0.31
Follow-up period (month)	41.0 (19.0, 63.0)	39.0 (21.0, 74.0)	0.66
Endoscopic follow-up period (month)	33.0 (9.0, 52.0)	25.0 (9.8, 51.3)	0.73
Synchronous tumors	2 (4.7)	25 (15.2)	0.077
Outcomes			
Death	3 (7.0)	11 (6.7)	0.77
Disease-specific death	0 (0.0)	0 (0.0)	1.00
Recurrence	0 (0.0)	0 (0.0)	1.00
Metachronous tumors	2 (4.7)	5 (3.1)	0.64

**Figure 2:**
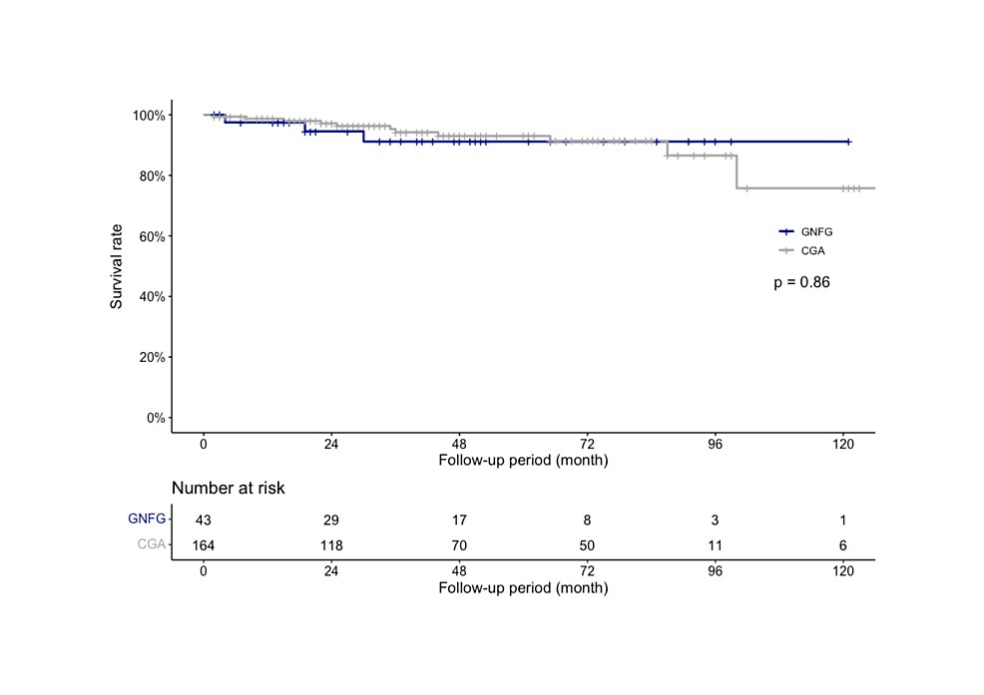
OS rate of GNFG and CGA after ESD The three-year OS rate was 91.2% in the GNFGs and 94.2% in the CGAs, which was not significantly different (P=0.86). OS: Overall survival; GNFG: Gastric neoplasm of the fundic gland type; CGA: Conventional gastric adenocarcinoma; ESD: Endoscopic submucosal dissection

The median follow-up period was 41.0 (19.0-63.0) months in the GNFG group and 39.0 (21.0-74.0) months in the CGA group, with 3 and 11 deaths, respectively. Although the cause of death was unknown in three cases in the CGA group, there were no primary deaths in either group. There was no significant difference in the OS between the two groups (P=0.86; Figure 2). In the GNFG group, the three- and five-year OS rates were both 91.2% (95% CI: 82.0-100%). In the CGA group, the three- and five-year survival rates were 94.2% (95% CI: 90.1-98.6%) and 93.0% (95% CI: 88.3-98.0%), respectively. No recurrence was observed in either patient group. The DSS and PFS rates were 100% in both groups.

**Figure 3:**
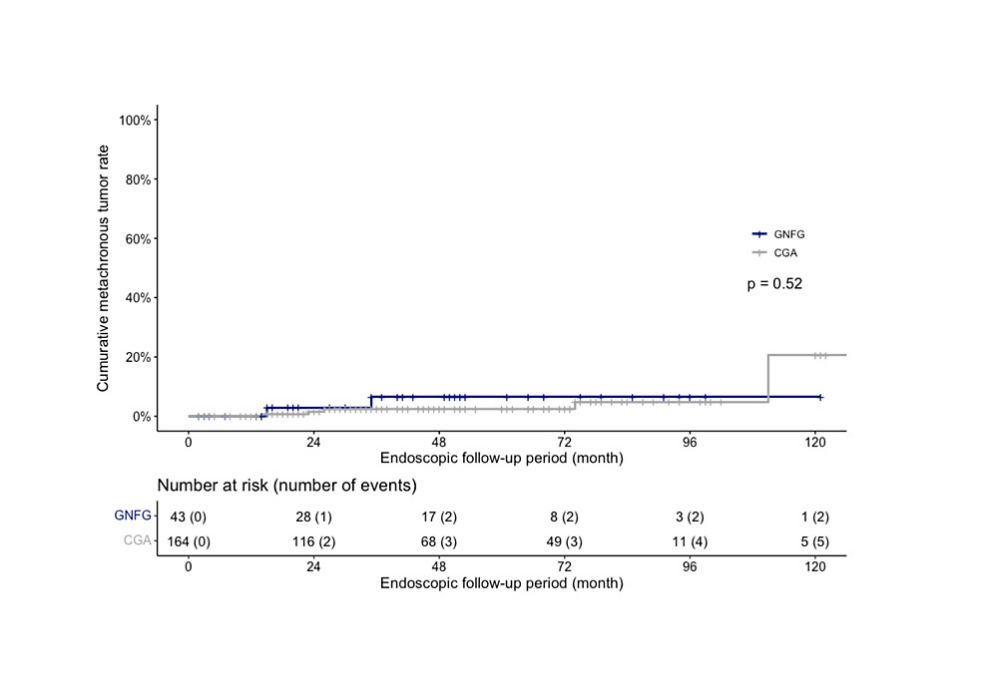
Cumulative metachronous tumor rate of GNFG and CGA after ESD The cumulative five-year metachronous gastric tumor incidence was 6.6% in GNFG and 2.5% in CGA, higher in GNFG but not significantly different (P=0.52). GNFG: Gastric neoplasm of the fundic gland type; CGA: Conventional gastric adenocarcinoma; ESD: Endoscopic submucosal dissection

The median endoscopic follow-up period was 33.0 (9.0-52.0) months in the GNFG group and 25.0 (9.8-51.3) months in the CGA group, respectively. The incidence of synchronous gastric tumors was lower in the GNFG group (4.7%) than in the CGA group (15.2%); however, the difference was not significant (P=0.077). The characteristics of synchronous gastric tumors are described in Table 2. Metachronous gastric tumors were observed in two cases in the GNFG group and five in the CGA group. The characteristics of metachronous gastric tumors are described in Table 3. There was no significant difference in the cumulative number of metachronous gastric tumors between the two groups (P=0.52; Figure 3). In the GNFG group, the cumulative three- and five-year metachronous gastric tumor rates were 6.6% (95% CI: 0.0-15.1%). In the CGA group, cumulative three- and five-year metachronous gastric tumor rates were 2.5% (95% CI: 0.0-5.2%).

**Table 2:** Characteristics of synchronous gastric tumors of GNFG and CGA Values are presented as n (%) and median (interquartile range). Continuous variables were compared using the Wilcoxon rank-sum test. For categorical variables, a Fisher's exact test was performed. Statistical significance was set at P<0.05. SM is classified into SM1 and SM2; SM1 is defined as cancer invasion <500 μm from the muscularis mucosae and SM2 is defined as invasion ≥500 μm. CGA: Conventional gastric adenocarcinoma; GNFG: Gastric neoplasm of fundic gland type; IQR: Interquartile range; M: Confined to mucosa; SM: Confined to submucosa

Variables	GNFG group N=2	CGA group N=26	P-value
Location			0.040
Upper third of the stomach	2 (100.0)	5 (19.2)	
Middle third of the stomach	0 (0.0)	9 (34.6)	
Lower third of the stomach	0 (0.0)	12 (46.2)	
Macroscopic type			0.056
Elevated	2 (100.0)	4 (15.4)	
Flat	0 (0.0)	7 (26.9)	
Depressed	0 (0.0)	13 (50.0)	
Mixed	0 (0.0)	2 (7.7)	
Histologic type			1.00
Differentiated	2 (100.0)	23 (88.5)	
Undifferentiated	0 (0.0)	3 (11.5)	
Tumor size (mm)	3.0 (2.5, 3.5)	10.0 (7.0, 15.5)	0.044
Tumor depth			0.071
M	1 (50.0)	26 (100.0)	
SM	1 (50.0)	0 (0.0)	
Tumor depth SM2	0 (0.0)	0 (0.0)	1.00
Ulceration findings	0 (0.0)	3 (11.5)	1.00
Lymphatic invasion	0 (0.0)	0 (0.0)	1.00
Venous invasion	0 (0.0)	0 (0.0)	1.00
Horizontal margin	0 (0.0)	2 (7.7)	1.00
Vertical margin	0 (0.0)	0 (0.0)	1.00

**Table 3:** Characteristics of metachronous gastric tumors of GNFG and CGA Values are presented as n (%) and median (interquartile range). Continuous variables were compared using the Wilcoxon rank-sum test. For categorical variables, a Fisher's exact test was performed. Statistical significance was set at P<0.05. SM is classified into SM1 and SM2; SM1 is defined as cancer invasion <500 μm from the muscularis mucosae and SM2 is defined as invasion ≥500 μm. CGA: Conventional gastric adenocarcinoma; GNFG: Gastric neoplasm of fundic gland type; IQR: Interquartile range; M: Confined to mucosa; SM: Confined to submucosa

Variables	GNFG group N=2	CGA group N=7	P-value
Location			0.083
Upper third of the stomach	1 (50.0)	1 (14.3)	
Middle third of the stomach	1 (50.0)	0 (0.0)	
Lower third of the stomach	0 (0.0)	6 (85.7)	
Macroscopic type			0.44
Elevated	2 (100.0)	3 (42.9)	
Flat	0 (0.0)	0 (0.0)	
Depressed	0 (0.0)	4 (57.1)	
Histologic type			1.00
Differentiated	2 (100.0)	7 (100.0)	
Undifferentiated	0 (0.0)	0 (0.0)	
Tumor size (mm)	5.0 (5.0, 5.0)	10.0 (7.0, 12.0)	0.13
Tumor depth			0.028
M	0 (0.0)	7 (100.0)	
SM	2 (0.0)	0 (0.0)	
Tumor depth SM2	0 (0.0)	0 (0.0)	1.00
Ulceration findings	1 (50.0)	0 (0.0)	0.22
Lymphatic invasion	0 (0.0)	0 (0.0)	1.00
Venous invasion	0 (0.0)	0 (0.0)	1.00
Horizontal margin	0 (0.0)	0 (0.0)	1.00
Vertical margin	0 (0.0)	0 (0.0)	1.00

## Discussion

We found no difference in OS between the GNFG and CGA groups. There was also no difference in the metachronous tumor rate. Therefore, it was not suggested that GNFG have a better prognosis than CGA or that they have a lower rate of metachronous tumors. Hence, it was suggested that GNFG, as well as CGA, may require post-treatment management according to current guidelines.

The lack of difference in OS between the two groups may be because there was no difference in the patient's general condition or comorbidities. This is because the OS could be assumed to have been influenced by the patient's general condition and comorbidities, as no tumor-related deaths were observed in either group. According to the results of multicenter studies in Japan, the age at the time of treatment was approximately 66 years for GNFG (the period covered was 2008-2019) [7] and approximately 72 years for early gastric cancer (the period covered was 2013-2016) [17], suggesting that GNFG has a younger onset than CGA, although not directly comparable. Therefore, in the present study, we adjusted the treatment date after matching for age and sex to reduce the effect on OS. There was no difference in the OS between the two groups, suggesting that GNFG does not have a better prognosis than CGA.

Although GNFG is considered a low-malignancy tumor, the rate of SM invasion is significantly higher in GNFG than in CGA. In this regard, GNFG is considered to have a high malignant potential. However, because of the small size of the GNFG, even if it invaded the submucosa, 93.8% (15/16) of the cases were eCura B after ESD and could be expected to be curative. Therefore, the prognosis may be similar to that of CGA when treated according to the curative criteria in the guidelines [15]. One case had eCura C-2; therefore, additional surgery was performed, and the patient was managed without recurrence or death. As there are few cases of non-curative ESD, future studies should assess lymph node metastasis and the prognosis of GNFG in eCura C-2. However, there is disagreement as to whether it is a carcinoma or adenoma with respect to intramucosal GNFG. In recent years, some reports have described intramucosal lesions as adenomas [6,7]. However, because intramucosal GNFGs are also differentiated and have small lesions, the endoscopic curability is eCura A if they are considered carcinomas. Therefore, even if the lesion is cancerous, it would meet the curative criteria. Therefore, it is difficult to evaluate whether there is truly no malignant potential and whether endoscopic treatment may have contributed to the improved prognosis of GNFG.

There was no significant difference in metachronous tumor rates, but the GNFG group had more metachronous tumors. We recognize that synchronous and metachronous carcinogenesis occurs in CGA. The synchronous and metachronous tumor rates have been reported to be 8.7-11% and 5.2-14% [18-20]. However, little is known about synchronous and metachronous tumor rates in GNFG. In particular, the metachronous tumor rate has not been reported. To the best of our knowledge, this is the first report of a metachronous tumor rate of 4.7%. Therefore, surveillance after ESD for GNFG is needed because metachronous tumors of GNFG are not as rare as those of CGA. The multiple lesion rate of GNFG was reported to be 5.3-11.1% [21-23], although none of the reports defined whether multiple lesions were synchronous or metachronous. The multiple-lesion rate of GNFG in this study was 13.9% (6/43) when synchronous and metachronous tumors were included. The multiple tumor rate is common in GNFG. Although image-enhanced endoscopy and magnifying endoscopy can increase the detection rate of early gastric cancer [24-26], no method has been reported to increase the detection rate of GNFG. Therefore, GNFG should usually be recognized by white light imaging but may be difficult to recognize because of its small size, even if it has endoscopic characteristics [4,21]. Therefore, we should be more aware of the possibility of multiple tumors, even when we detect them.

This study had several limitations. First, this was a single-center retrospective study, and bias was inevitable. Second, the few cases with eCura C2 may have underestimated the malignant potential of GNFG. Last, this study adjusted for age, sex, and treatment data, but not for other factors, owing to the limited sample size. Despite these limitations, to the best of our knowledge, this study has the advantage of comparing the prognosis and metachronous tumor rates of GNFG and CGA, which will contribute to the future management of GNFG.

## Conclusions

The prognosis for GNFG is good, but not better than that for CGA. The metachronous tumor rate in GNFG was not lower than that in CGA. Therefore, the surveillance after treatment of GNFG may need to be the same as that for CGA.
